# The role of Antibody Vκ Framework 3 region towards Antigen binding: Effects on recombinant production and Protein L binding

**DOI:** 10.1038/s41598-017-02756-3

**Published:** 2017-06-19

**Authors:** Chinh Tran-To Su, Wei-Li Ling, Wai-Heng Lua, Jun-Jie Poh, Samuel Ken-En Gan

**Affiliations:** 1 Bioinformatics Institute, Agency for Science, Technology and Research (A*STAR), Singapore, Singapore; 2 p53 Laboratory, Agency for Science, Technology and Research (A*STAR), Singapore, Singapore

## Abstract

Antibody research has traditionally focused on heavy chains, often neglecting the important complementary role of light chains in antibody formation and secretion. In the light chain, the complementarity-determining region 3 (VL-CDR3) is specifically implicated in disease states. By modulating VL-CDR3 exposure on the scaffold through deletions in the framework region 3 (VL-FWR3), we further investigated the effects on secretion in recombinant production and antigen binding kinetics. Our random deletions of two residues in the VL-FWR3 of a Trastuzumab model showed that the single deletions could impact recombinant production without significant effect on Her2 binding. When both the selected residues were deleted, antibody secretion was additively decreased, and so was Her2 binding kinetics. Interestingly, we also found allosteric effects on the Protein L binding site at VL-FWR1 elicited by these deletions in VL- FWR3. Together, these findings demonstrate the importance of light chain FWR3 in antigen binding, recombinant production, and antibody purification using Protein L.

## Introduction

Both the antibody light (L) and heavy (H) chains contain constant (C) and variable (V) regions^1^, the latter V-regions, are further characterized into framework regions (FWRs) and complementarity-determining regions (CDRs)^2, 3^. The FWRs of both chains are predominantly β-sheets, and come together to support the CDRs hypervariable loops that further interact with each other to confer antigen specificity^4^.

Of the CDRs, VH-CDR3 has marked influences on antigen specificity^5–10^, making VH regions the typical focus for antigen-specificity. Nonetheless, circulating light chains, which play a complementary role in antigen recognition^11, 12^, are also implicated in diseases^11–13^, in particularly VL-CDR3 in autoimmunity diseases such as rheumatoid arthritis^14^. Large-scale analyses of antibody sequences^15, 16^ and structures^17, 18^ showed that key residues in the FWRs, stabilized the antibody structure and can play an allosteric contributory role in antigen binding. Random framework mutation studies also demonstrated that certain residue positions can allosterically influence the packing of antigen-binding regions^15, 18, 19^. For example, point mutations in the FWRs can improve the affinity of grafted CDRs towards the antigen^20^.

In order to further study the importance of Vκ-FWR3 on antigen (Her2) specificity, recombinant production, and protein L binding, we incorporated two random deletions in the Vκ-FWR3 of a recombinant Trastuzumab model to reduce Vκ-CDR3 exposure. In this, we aim to further investigate the contribution of VL-FWR3 (without directly manupulating VL-CDR3) to antibody secretion in recombinant production, protein L binding, and most importantly, antigen binding, which will influence CDR grafting and antibody humanization.

## Results and Discussion

**Figure 1:**
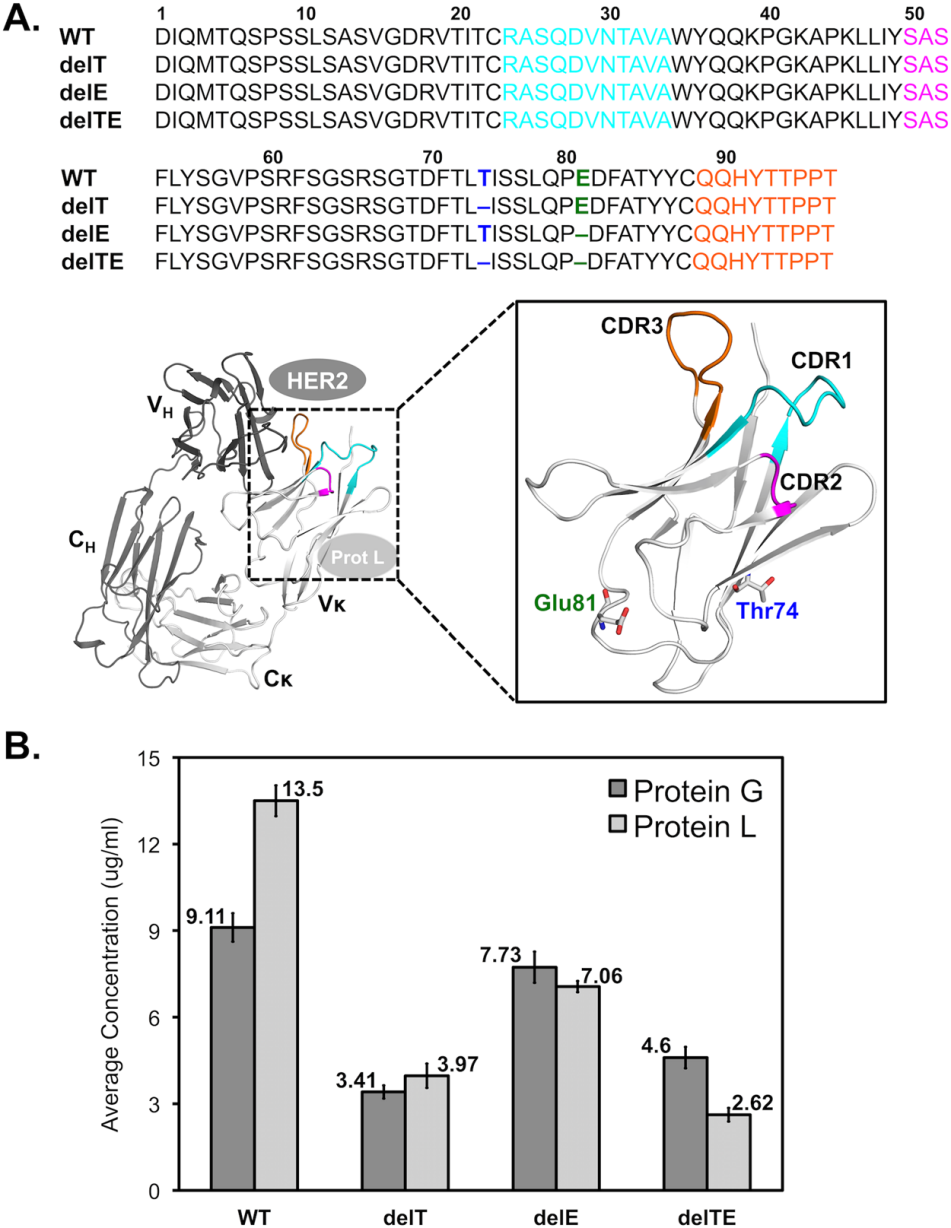
The Trastuzumab mutants (WT, delT, delE, delTE) used in this study (**A**) Sequence alignments of the Trastuzumab mutants with CDRs highlighted as follows: Vκ-CDR1 (cyan), Vκ-CDR2 (magenta), and Vκ-CDR3 (orange). The deleted residues T74 and E81 are in blue and green, respectively. The structure of the WT-Fab region is shown in dark gray (heavy chain) and in light gray (κ light chain). Schematics of Her2 binding (at CDRs) and protein L binding (at Vκ-FWR1) are shown. (**B**) Antibody secretion levels of Trastuzumab mutants as determined by Protein G and L biosensor. Each bar shows the average concentration (μg/ml) of Trastuzumab WT and mutants in cell culture supernatants from three independent experiments using protein G and protein L biosensors, followed by statistical analysis using ANOVA and two tailed T-Test. The ANOVA and T-test results showed that the mutants are significantly different from one another (*p < *0.05) within each biosensor data set (i.e. Protein L or G).

We set out to investigate the effect of Vκ-FWR3 manipulation on Vκ-CDR3, recombinant antibody production, allosteric effects to Protein L binding, and antigen binding. We performed only up to two random deletions in the Vκ-FWR3 as our computational studies showed that no more than two residues should be deleted in the Vκ-FWR3 to maintain optimal structural stability in the light-heavy chain interface (Supplementary Fig. S2). The criteria for guiding the random deletions were that the mutations must be in the FWR3, but not flanking the CDR3 to prevent direct effects on CDR functions. Since the hydrophobic core residues at the intra-domain region are involved in stabilizing the antibody structure and might affect antigen binding^15, 18^, we decided to pick one of the deletions within this region (Vκ-FWR3 residues 57–88). Since the core region is formed by three β strands (residues 62–67, 70–75, 85–88), of which the former two are involved in forming the intra-domain core, we avoided selecting the conserved residues^15^ and all the lower and upper core residues (i.e. buried residues). Of the remaining surface residues: S63, S65, D70, T72, and T74, the residue T74 was found the least conserved^15^ in consensus profile of the Natural Sequence Database and in between two conserved residue of the extended core (L73 and I75)^18^, thus we selected T74 for deletion. The other deletion of E81 was chosen at random between the two exposed residues (A80 and E81) in the loop region that bridged the two β strands above. The resulting Trastuzumab mutants are: deleted T74 (delT), deleted E81 (delE), and double deleted mutant (delTE) (shown in Fig. 1A).

To measure the effect of antibody secretion of recombinant production of these Trastuzumab mutants (co-transfected with Trastuzumab heavy chain plasmid), we first used protein G biosensors (which binds to the IgG heavy chain). Protein G biosensor analysis of the transfected cell culture supernatants (Fig. 1B) showed the wild-type production (9.11 ± 0.49 µg/ml) to be the highest and that all production are significantly different from one another [F(3,8) = 116.33, p < 0.01] using ANOVA. When comparing production against wild-type, all mutants were significantly different with delT at 3.41 ± 0.23 µg/ml [t(4) = −18.08, p < 0.01], delE at 7.73 ± 0.54 µg/ml [t(4) = −3.26, p < 0.05] and delTE at 4.60 ± 0.37 µg/ml [t(4) = −12.63, p < 0.01] using two-tailed T-test with 95% confidence. Since the two random deletions are distant from the cysteine sites (residues C214 and C437) forming the disulfide bridge between the light and heavy chains, this decrease in antibody secretion may be a result of internal steric hindrances during domain folding. Since position 74 substitutions did not cause significant effect in a previous study^15^, the production decrease in delT, which was lower than that of delTE, was surprising. Hence, the E81 deletion may partially compensate for the structural effects of Vκ-FWR3 delT in recombinant antibody secretion.

**Figure 2:**
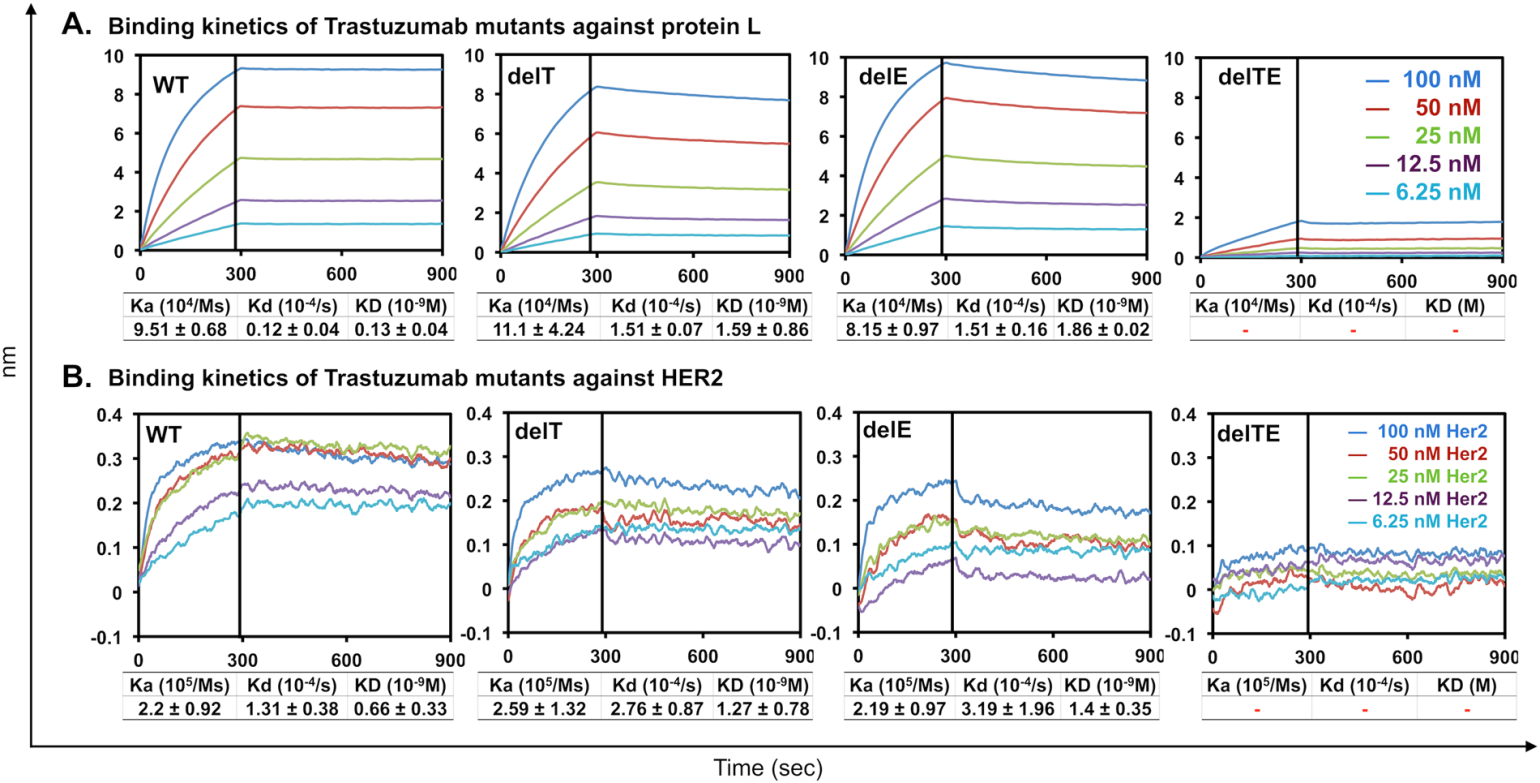
Binding kinetics profiles of Trastuzumab WT and its mutants from three independent experiments with corresponding average association (Ka), dissociation (Kd) and overall binding kinetic (KD) values with standard deviations. (**A)** Binding kinetics of decreasing concentration (100 nM to 6.25 nM) of Trastuzumab mutants to protein L biosensor, and (**B**) binding kinetics of Trastuzumab mutants using anti-human Fc capture biosensor to varying concentrations of Her2 (100 nM to 6.25 nM). Poor binding responses of the delTE mutant against protein L and Her2 produced unreliable binding kinetics values as determined by the Octet software for response value < 0.1.

Initially used for quantification validation of Protein G results, our Protein L (which binds to Vκ-FWR1) quantification results showed significant discrepancies in production levels of wild-type at 13.5 ± 0.53 µg/ml [t(4) = −10.19, p < 0.01] and for delTE at 2.62 ± 0.23 µg/ml [t(4) = 7.85, p < 0.01] than those analyzed by Protein G biosensor (Fig. 1B). We further investigated the binding kinetics of the Transtuzumab mutants to protein L and found that the double deletions decreased protein L binding in terms of binding capacity (Fig. 2A). On the other hand, the single deletions only reduced Protein L binding kinetics but not binding capacity, where KD values of delT and delE were 1.59 ± 0.86 × 10^−9^ M and 1.86 ± 0.02 × 10^−9^ M, respectively, as compared to that of wild-type (KD value of 0.13 ± 0.04 × 10^−9^ M). The differences were derived from the dissociation rate K_d_ (Fig. 2A) where both delT (K_d_ ~ 1.51 ± 0.07 × 10^−4^ s^−1^) and delE (K_d_ ~ 1.51 ± 0.16 × 10^−4^ s^−1^) showed 12-fold higher rates than the wild-type (K_d_ ~ 0.12 ± 0.04 × 10^−4^ s^−1^). These findings suggest that the single deletions elicited effects that resulted in faster dissociation without significantly affecting the association to protein L binding.

**Figure 3:**
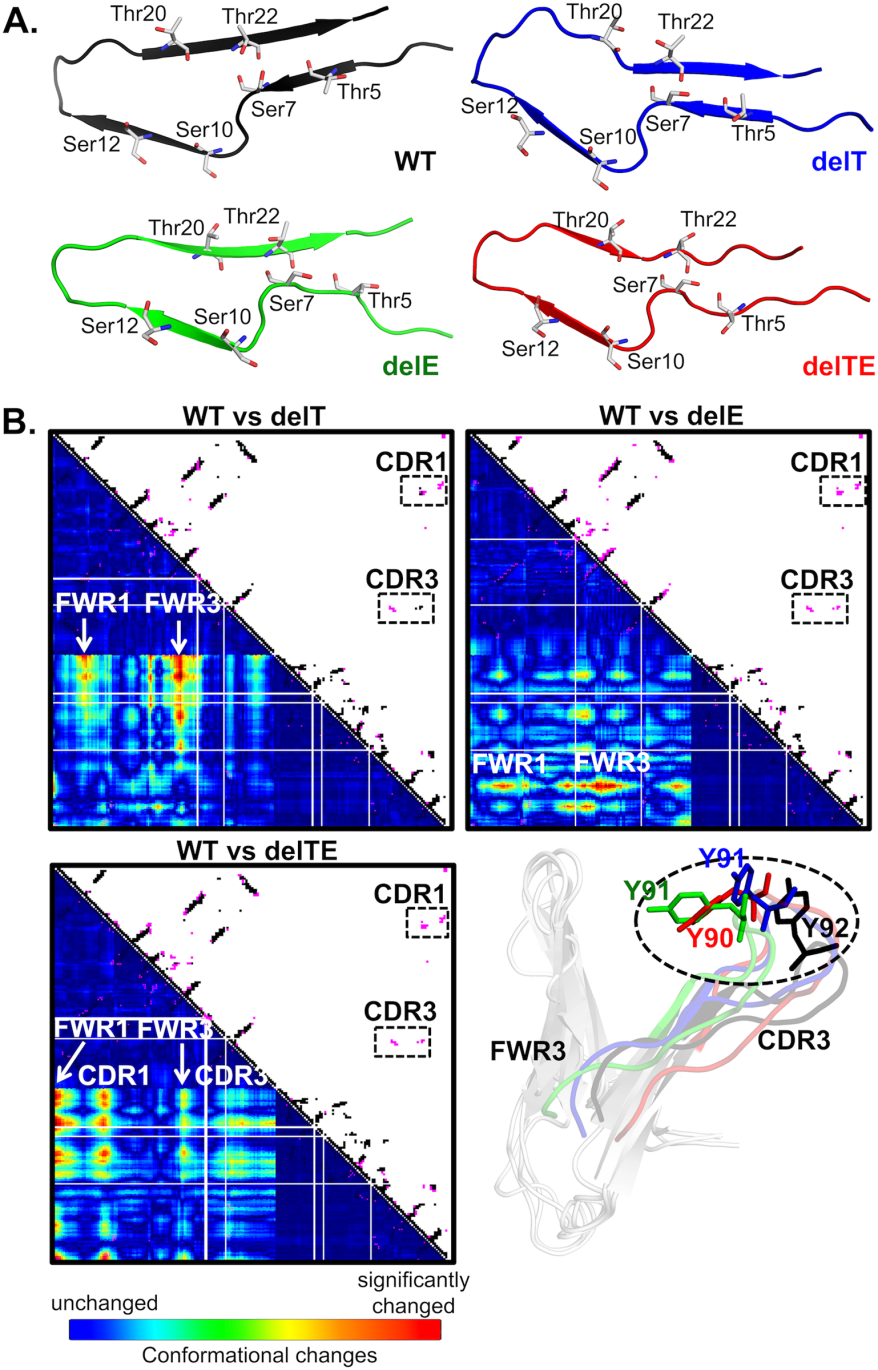
Results of the structural analyses of the Trastuzumab mutants FWRs and CDRs. (**A**) Damages in the Vκ-FWR1 β-strands (residues 5–22) subsequently caused conformational changes in the interacting regions and diminished the binding of protein L (Figs S3 and S4). (**B**) Contact maps of the Fab mutant-Her2 complexes against the Fab WT-Her2 complex showed common interactions (black dots) and WT-only interactions (magenta dots). Vκ-CDR1 and Vκ-CDR3 contacts with Her2 are shown in the boxes. Conformational changes of the Vκ-CDR3 loop with different orientations of Y92^WT^ are shown (exposure is shown in Fig. S5B of Supplementary Materials). The mutants are colored as in (A): WT (black), delT (blue), delE (green) and delTE (red). All models were made using I-TASSER with PDB: 1n8z as template and minimized using AMBER14.

To investigate this further, we performed structural modeling of the mutants to study the effects on protein L binding. Since protein L binds to Vκ-FWR1 domain^21, 22^ via a formation of β-zipper interactions^23^, particularly between its β_2_-strand and β-strands of the Vκ-FWR1 domain (residue 5–12), the interaction is highly dependent on the backbone conformations of the two interacting partners that results in the burying of solvent accessible areas at the interface^23^. Our models showed that the deletions damaged the Vκ-FWR1 β-strands (residues 5–7, 10–12, 20–22) through conformational changes of the Vκ-FWR1 domain that reduced the accessibility of those interacting β-strands (Fig. 3A and Supplementary Figs S3 and S4). When compared to the wild type structure, deleting both T74 and E81 caused synergistic burying of the anti-paralleled β-strands to result in unfavorable binding modes of protein L (Supplementary Fig. S3). Such changes may be useful to engineer antibodies that would be inert to superantigens such as protein L that may otherwise elicit unwanted effects^24–26^ in therapy while retaining antigen-binding properties of the antibodies.

In the most important test of binding kinetics to the known Her2 antigen using Anti-Human Fc capture (AHC) biosensors (Fig. 2B), we found the single deletion mutants: delT and delE, retained good binding kinetics at KD values of 1.27 ± 0.78 × 10^−9^ M and 1.4 ± 0.35 × 10^−9^ M, respectively. These were only slightly lower than the wild type (KD value of 0.66 ± 0.33 × 10^−9^ M). On the other hand, the delTE mutant demonstrated very poor binding (Fig. 2B) with no reliable readouts. To explain this, our contact maps (Fig. 3B) of overlapping interactions between the mutant/Her2 complexes and the wild-type/Her2 complex revealed that several key contacts between Vκ-CDR1/CDR3 and Her2 were absent in all the mutant complexes. The single deletion mutants retained interactions with Her2 via Vκ-CDR3 and/or Vκ-CDR1 despite conformational changes in Vκ-FWR3 (Fig. 3B and Supplementary Fig. S5A); however, these interactions were lost in the double deletion mutant. Interestingly, our results of the single deletion mutants did not support previous random framework mutation studies that showed effects on antigen-binding^15, 18–20^, rather it was only the double deletion that showed significant binding kinetics differences. This may be due to the nature of the manipulation (where we used deletion and the other studies typically used substitutions), and also the nature of the antibody.

Nonetheless, we observed that the exposure of Vκ-CDR3 residue (Y92^WT^) to Her2 decreased in all the mutants due to the reduced Vκ-FWR3 lengths (Supplementary Fig. S5B). In the single deletion mutants, the Y92 side chain bent inwards towards the Vκ-FWR3, causing conformational changes in the Vκ-CDR3 loop (more pronounced in delTE than in delE in Fig. 3B), hence decreasing contacts with Her2. The majority of the anti-parallel β-strands (forming the core to hold up the Vκ-CDR3) were found to become coils. Deleting T74 alone caused significant damages in these β-sheets (residues 62–67 and 70–75) and the double deletion of residues T74 and E81 caused additive damages in these regions (see Supplementary Fig. S5A, where majority of disordered structure or coils were seen in our dynamics simulation). On the other hand, deleting the single E81 caused only a slight distortion in these regions given that it was not in the β-sheet core (Fig. 1B).

In conclusion, our study demonstrated that the Vκ-FWR3 scaffold could affect Vκ-CDR3 exposure to protein L and Her2. While this may give rise to novel therapeutic antibodies with reduced responses to superantigens (e.g. protein L), there are undesirable effects on recombinant production. Our work further advocates the complementary role of light chain in overall antigen recognition, particularly in antigen dissociation. While our study also demonstrated that more than one deletion in the Vκ-FWR3 could as well abolish antigen binding, extended structural studies are required before we can fully understand the intricacies involved in CDR grafting and antibody secretion.

## Materials and Methods

### Cloning of Trastuzumab mutants

Based on a previous recombinant Trastuzumab variant^27^, Trastuzumab mutants of single deletions del74T (delT), del81E (delE), and combined deletions (delTE) were generated using site-directed mutagenesis (Cat no: 200522, Agilent Technologies) using the following primers: del74T_For: 5′-CTG-ATT-TTA-CTC-TTA-TTT-CTT-CTC-TTC-AAC-C-3′; del74T_Rev: 5′-GGT-TGA-AGA-GAA-GAA-ATA-AGA-GTA-AAA-TCA-G-3′; and del81E_For: 5′-CTT-CTC-TTC-AAC-CTG-ATT-TTG-CTA-CTT-3′; del81E_Rev: 5′-AAG-TAG-CAA-AAT-CAG-GTT-GAA-GAG-AAG-3′. To create delTE, del74T plasmid was further mutated using the del81E primer set.

### Production of recombinant proteins

The plasmids were used for transient transfection as previously described^28^ with minor adjustments (1:1 light: heavy chain ratio, transfection mix at 5% v/v to DMEM with 10% low IgG FBS, Pan Biotech). Monomeric fractions of the antibodies (Supplementary Fig. S1) were obtained after Protein G affinity chromatography and size exclusion (Superdex 200 pg 16/600) using AKTA Pure system (GE healthcare). Concentrated antibodies (using 100 KDa Amicon Ultra protein concentrator, Cat no: UFC910096, Merck Millipore) were quantified (Nanodrop 1000 spectrophotometer, Thermo Fisher Scientific; and calculated protein coefficients) and analyzed using SDS-PAGE. Band sizes (Supplementary Fig. S1) were determined as previously described^29^.

### Quantification and kinetics measurement

Antibodies in culture supernatants were measured using Protein G (Cat no: 18-5082, Fortebio, Pall) and Protein L (Cat no: 18-5085, Fortebio, Pall) biosensors in the Octet QK^e^ system (Cat no: 30-5046, Fortebio, Pall) using pre-loaded programs (high sensitivity assay with regeneration) in the Octet data acquisition 7.0. Binding kinetics to Protein L was measured by direct binding of purified antibodies from 100 nM to 6.25 nM using 1X kinetic buffer (Cat no: 18-1092, Fortebio, Pall) with the following settings: pre-conditioning of protein L biosensor- three repeated runs of 0.2 M glycine, pH 2.5 for 20 secs followed by 1x kinetic buffer for 20 secs; baseline- 1x kinetic buffer for 120 secs; association of antibodies- 100 nM to 6.25 nM for 300 secs; dissociation of antibodies- 1x kinetic buffer, 600 secs; regeneration of protein L biosensor- three repeated runs of 0.2 M glycine, pH 2.5 for 5 secs followed by 1x kinetic buffer for 5 secs. The entire process from initial baseline to regeneration of protein L biosensor was repeated up to four times for kinetic testing of the four different antibodies in a single run. For Her2 binding kinetics, purified antibodies were first bound to Anti-Human Fc Capture biosensors (Cat no: 18-5060, Fortebio, Pall) prior to measurements to Her2 (100 nM to 6.25 nM, Cat no: H10004-H08H, Sino Biologicals Inc.) with the following settings: pre-conditioning of AHC biosensor- three repeated runs of 0.2 M glycine, pH 2.5 for 20 secs followed by 1x kinetic buffer for 20 secs; initial baseline- 1x kinetic buffer for 120 secs; loading of antibodies (with threshold set at 1 nm signal change and minimum 30 secs filtering), 20 nM for 300 secs; baseline- 1x kinetic buffer for 120 secs; association of Her2- 100 nM to 6.25 nM for 300 secs; dissociation of Her2- 1x kinetic buffer for 600 secs; regeneration of AHC biosensor- three repeated runs of 0.2 M glycine, pH 2.5 for 5 secs followed by 1x kinetic buffer for 5 secs. The entire process from initial baseline to regeneration of AHC biosensors were repeated for up to four times for kinetic testing of four different antibodies in a single run.

### Statistical analysis

The triplicate results obtained from quantification of the wild type and its mutants using protein G and protein L were analysed using both ANOVA, single factor test and two tailed T-Test, two samples assuming equal variances in Microsoft Excel 2007.

### Structural modeling of Trastuzumab mutants

The Fab models of the Trastuzumab mutants were constructed using I-TASSER^30^ using the PDB:1N8Z as template. The Fc was modelled from the FcγRIIa-IgG1 complex (PDB:3RY6). All models were minimized prior to analyses. Implicit solvent molecular dynamics simulations was used with random velocities (2 × 300 ns employing the Generalized Born model in the AMBER14 packages^31^ with force field ff12SB). During the simulation, the Langevin temperature equilibration scheme (with collision frequency of 1 ps^−1^) was used to maintain the simulation at 300 K for every 2 fs time step, and SHAKE algorithm was applied to constrain bonds that involved hydrogen.

### Protein docking of Trastuzumab mutants to protein L and Her2

Representatives of the four mutants were selected using the median of FWR1 backbone RMSD from the full trajectories. The FWR1 backbone RMSD was calculated against PDB:1IGY structure. The one-armed models were dimerized using the full-length IgG1 structure PDB:1IGY as the backbone template. Docking to Protein L and Her2 by the mutants (using Vκ-FWR1 residues 5–12, 18, 20, 22, 24 that are known to interact with protein L^23^; CDR1 residues 24–34 on Vκ and residues 240–249 on VH, and CDR3 residues 89–96 on Vκ and residues 311–323 on VH that interact with Her2^32^) were performed using HADDOCK server^33^. The most populated and highest HADDOCK scoring complexes ranked at the top cluster were used for subsequent analyses. Contact maps were constructed and analyzed using CMView v1.1^34^, and visualizations were generated using PyMOL v1.4.1^35^.

### Ethics approval

No animal or human primary tissues or cells were used in this study.

## Electronic supplementary material

Supplemenatary Materials
