# Supplementary material for: The role of Antibody Vκ Framework 3 region towards Antigen binding: Effects on recombinant production and Protein L binding
Source: Sci Rep. 2017 Jun 19;7:3766. doi: 10.1038/s41598-017-02756-3 (PMC5476676; doi:10.1038/s41598-017-02756-3)
Supplement: Supplementary file 1 — Supplemenatary Materials [file 41598_2017_2756_MOESM1_ESM.pdf]

The role of Antibody Vκ Framework 3 region towards Antigen binding: Effects on recombinant production and Protein L binding.

Chinh Tran-To Su[1#], Wei-Li Ling[1#], Wai-Heng Lua[1], Jun-Jie Poh[1], Samuel Ken-En Gan[1,2*]

Affiliation:

[1]Bioinformatics Institute, Agency for Science, Technology and Research (A*STAR), Singapore

[2]p53 Laboratory, Agency for Science, Technology and Research (A*STAR), Singapore

[#]Co-first author

[*]Correspondence author: samuelg@bii.a-star.edu.sg

Bioinformatics Institute, A*STAR

30 Biopolis Street, #07-01 Matrix

Singapore 138671

Tel: +65 6407 0584

Fax: +65 6478 9047

Supplementary 1

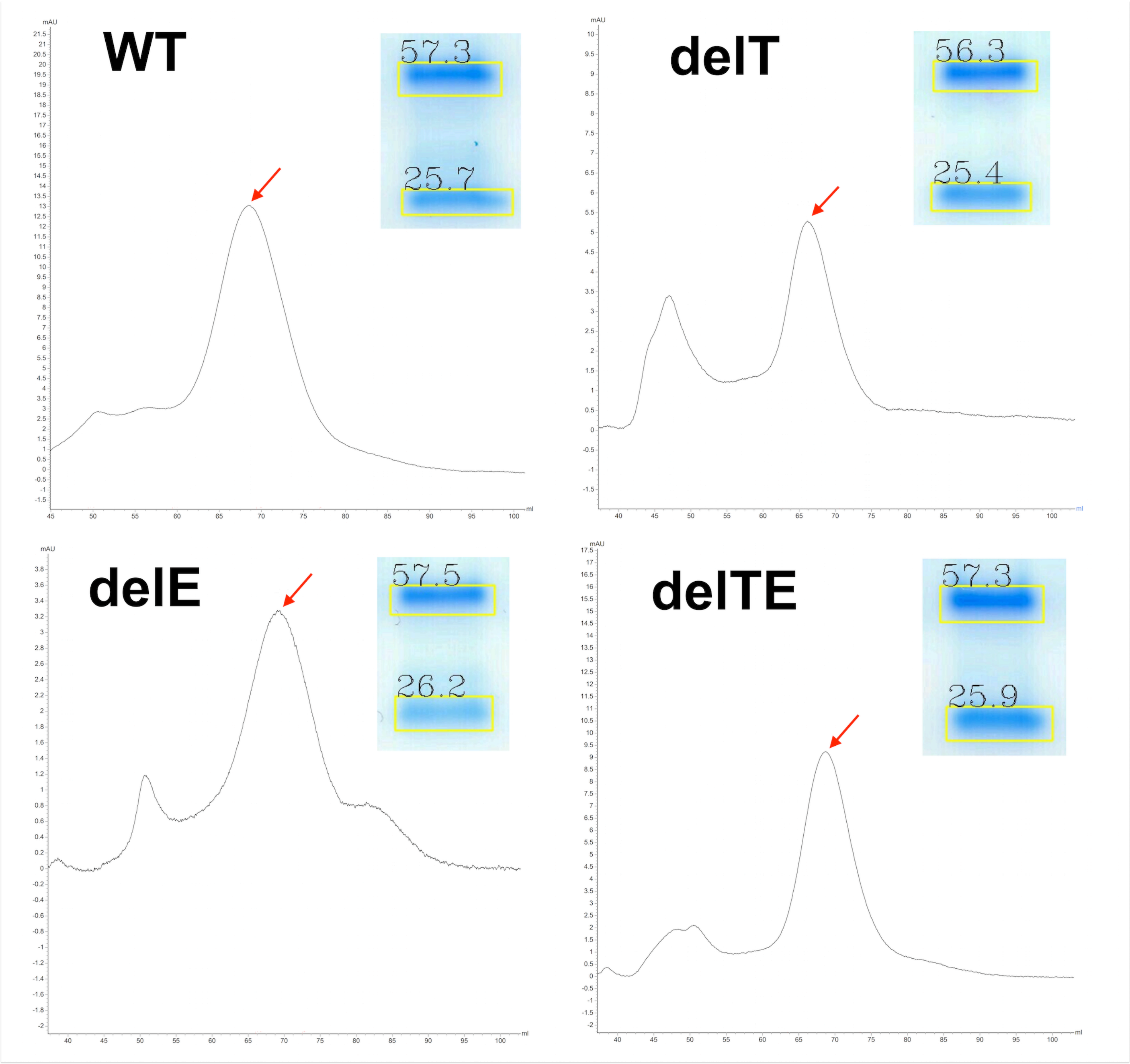

Figure S1: Size exclusion chromatography of purified Trastuzumab mutants and their corresponding reducing SDS-PAGE analysis with band sizes determined by GelApp. X-axis shows time in minutes and the Y-axis shows the UV absorbance in mAU.

Supplementary 2

To evaluate changes in structural stability of the wild type Trastuzumab Fab region, free energy (ΔΔG) and vibrational entropy (ΔS) were estimated using the ENCoM server[1]. The interface between the light-heavy chains (identified using PDB: 1N8Z) were mutated accordingly to *N* random deletions *(N = 1,…,10)* in the FWR3.

We defined the level of stability damages when the number of deletions increases from *i* to *j* as below:

$$\text{Damage (\%)} = \frac{|(\Delta\Delta G_i - \Delta\Delta G_j)|}{\Delta\Delta G_j}$$

Therefore, according to our analysis (Figure S2), the structures with N= 2, 5, or 8 deletions maintain better stabilities (more favorable ΔΔG). With the highest cost of instability when increasing numbers of deletions (most damaged if an extra residue is removed), it was implied that 2 deletions might be the maximum numbers of deletions at the FWR3 that the Trastuzumab Fab could maintain the optimal structural stability.

Hence, we selected *N=2* as the number of deletions that would be further explored after the single deletions were performed.

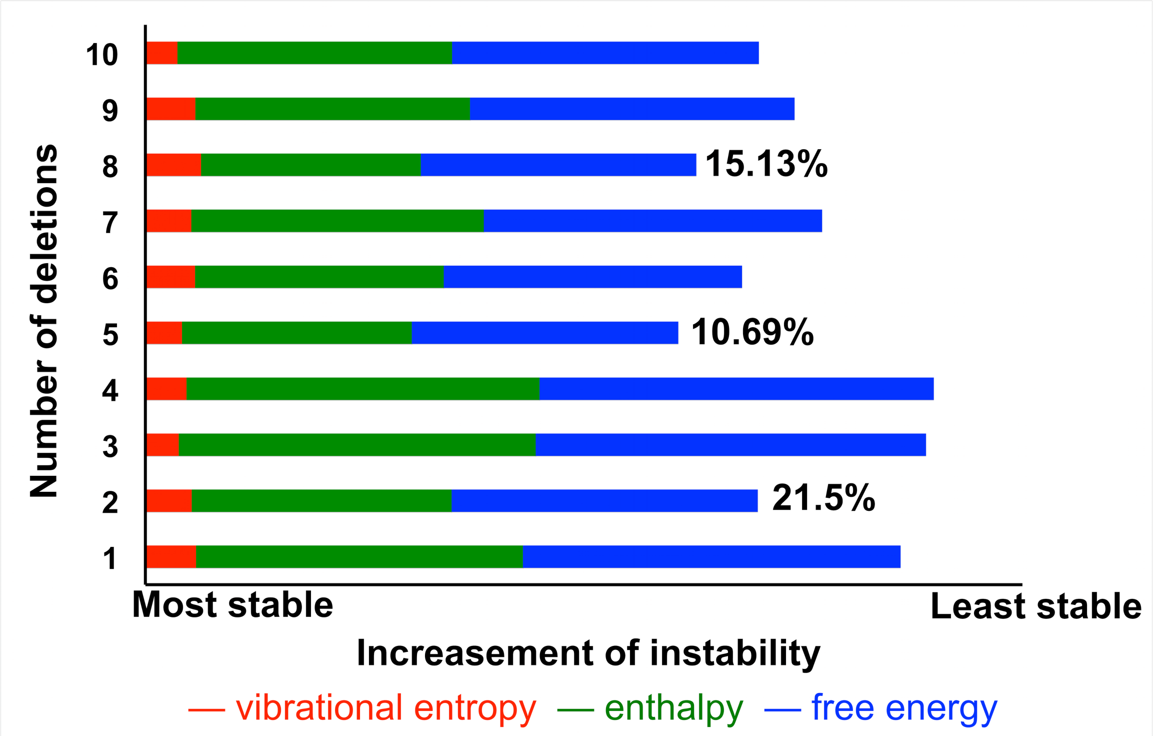

Figure S2: Analysis of structural stability of the Fab Trastuzumab for different numbers of deletions. The shown percentage values represent level of damages (in terms of structural stability using free energy) when the number of deletions increases. For examples in the case of 2 deletions, the Fab structure would lose 21.5% stability when the third residue was removed from the FWR3.

1. Frappier V, Chartier M, Najmanovich RJ. ENCoM server: exploring protein conformational space and the effect of mutations on protein function and stability. Nucleic Acids Res 2015; 43:W395-W400

Supplementary 3

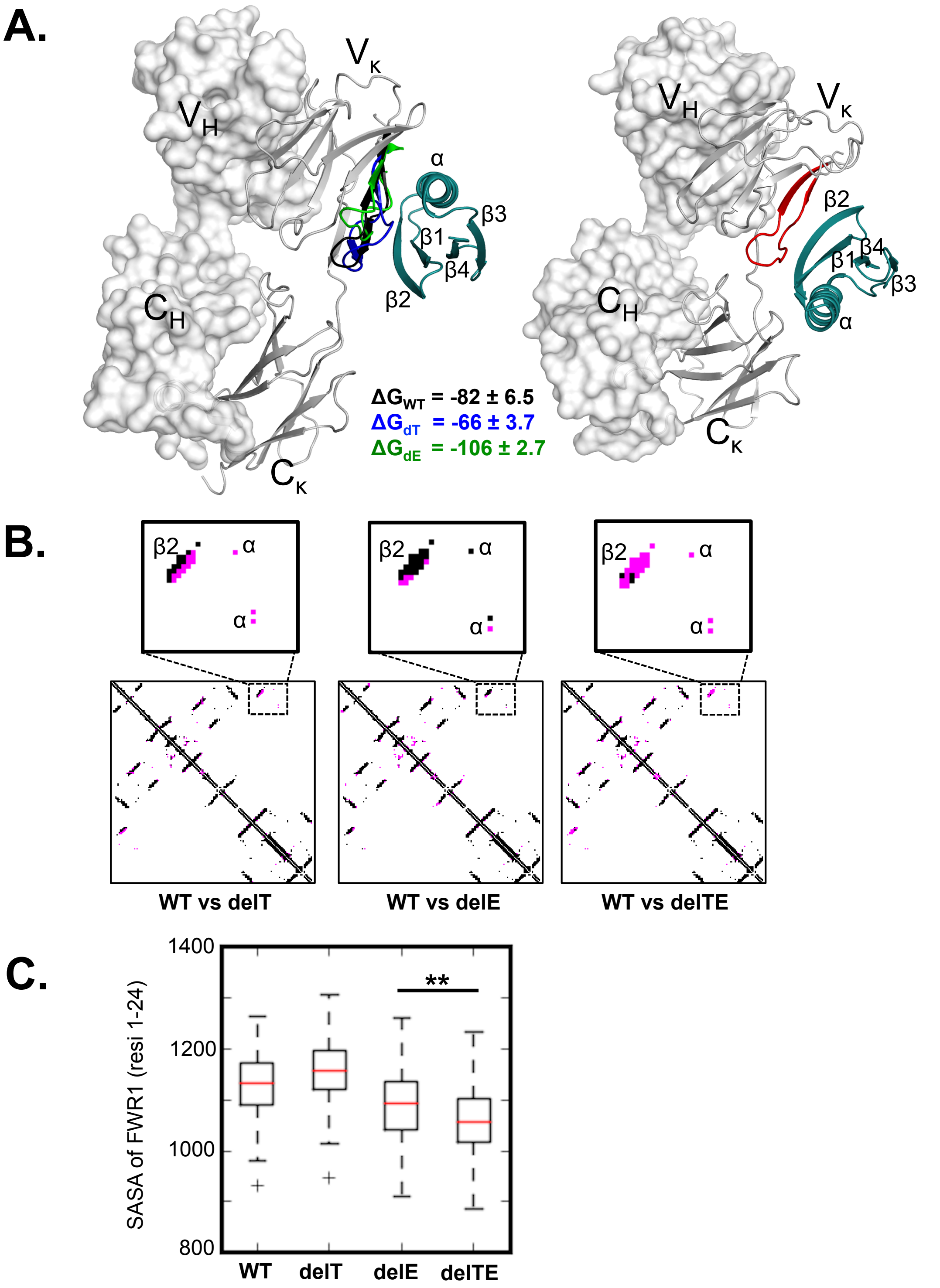

Figure S3: (A). Unfavorable binding mode of protein L to the delTE mutants when compared to WT, in which the protein L flipped 180 degree outward from the signature binding mode (Graille et al. 2001) to the Vκ-FWR1 of Trastuzumab. The β strands involved in the interactions are highlighted in black (WT), blue (delT), green (delE), and red (delTE). Protein L (deep teal color) contains 1 helix and four β-strands (β2 interacts with Vκ-FWR1). Protein L binding energies of the WT and of the two single mutants were estimated using MMGBSA method in AMBER14 package. (B) Contact maps of the 3 mutants superimposed on that of WT highlighting diminishing binding effect to protein L (at β2 strand and α helix). (C) Exposures of the Vκ-FWR1 (residue 1-24) were estimated using solvent accessible surface area (SASA) by NACCESS. The discrepancy of the SASA between delT and delTE were confirmed using a two tailed T-test with **p-values < 0.0005 (with 95% confidence).

Supplementary 4

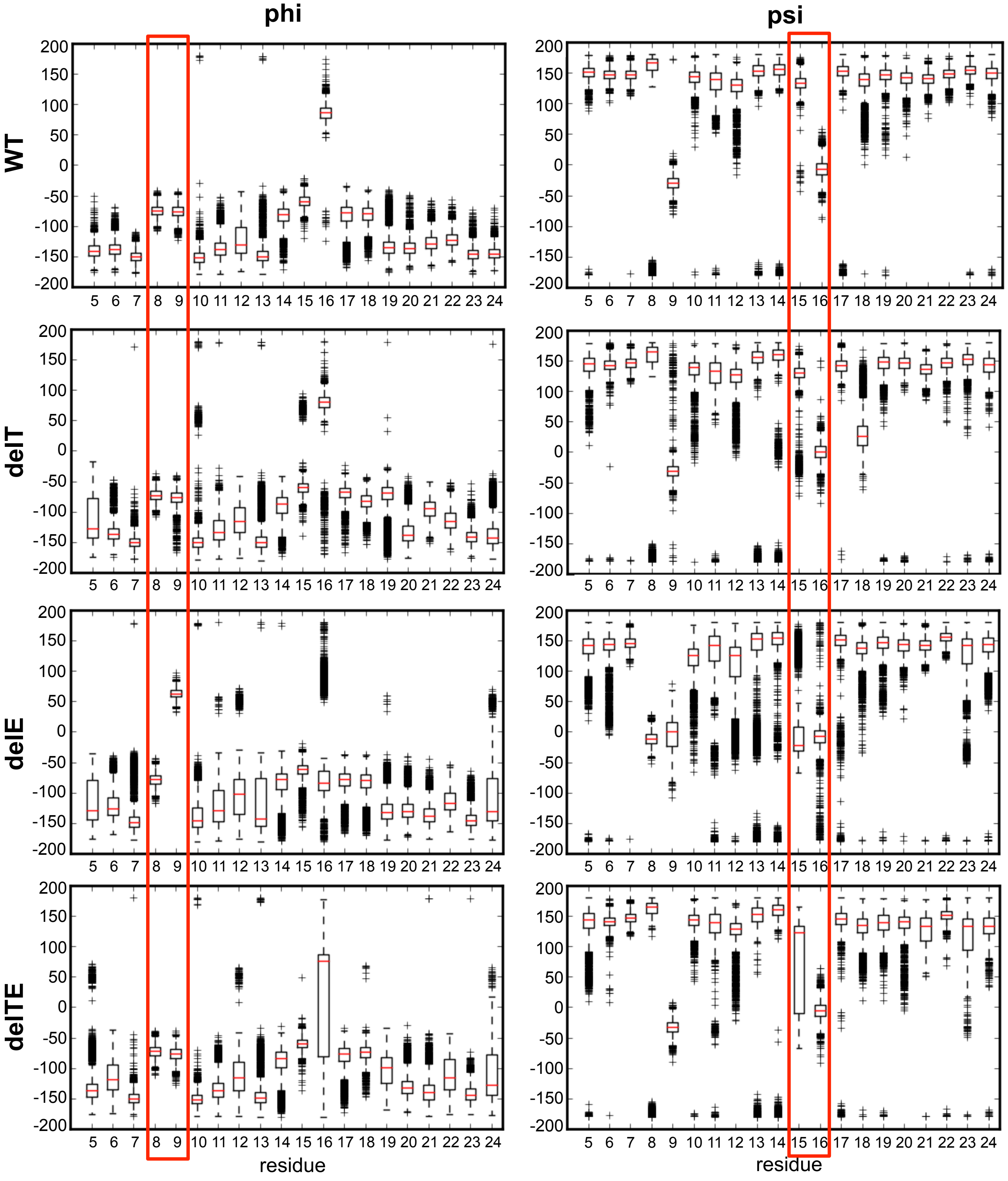

Figure S4: Dihedrals of Vk-FWR1 (particularly at residues 5-24) during 600ns molecular dynamics simulation. It shows that the backbone conformations of this region in the mutants have substantial changes (e.g. beta-turns at residues 8-9 and 15-16) as compared to the WT. This may consequently affect the protein L binding, which was known to be highly dependent on main chain conformation of the antibody (Graille et al. 2011).

Supplementary 5

A.

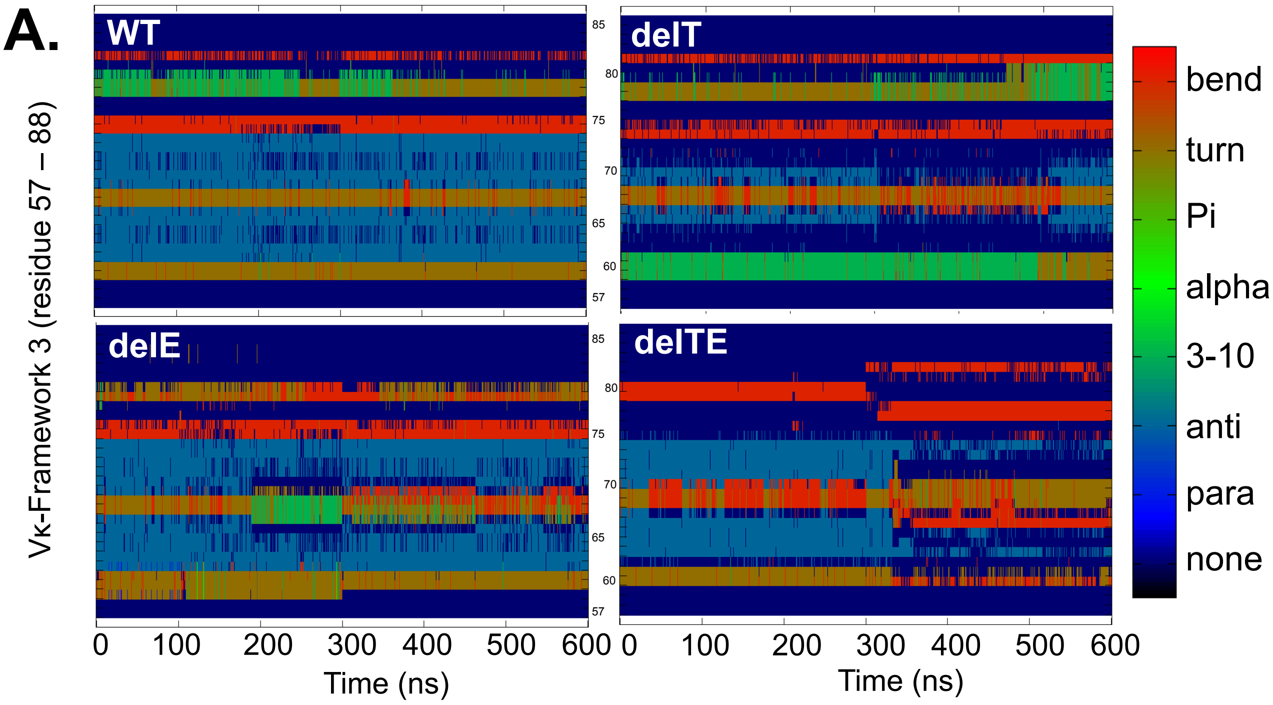

B.

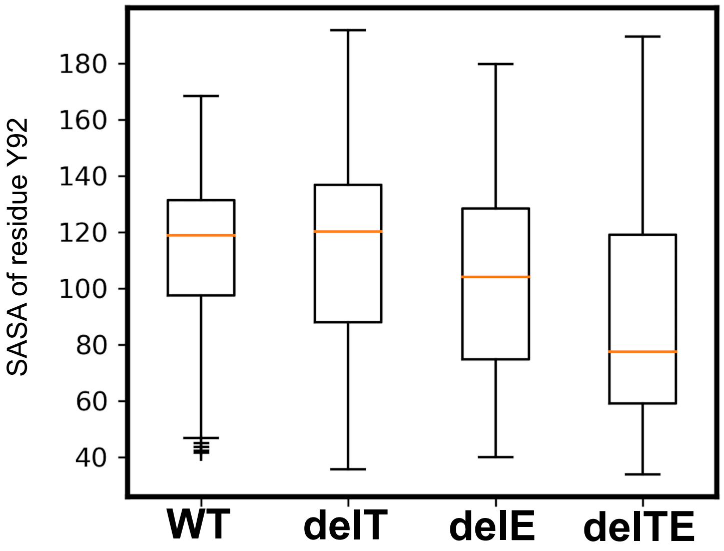

Figure S5: (A). Secondary structure analysis of the variant Vκ-FWR3 using the program DSSP implemented in AmberTools14. (B) Exposures of the residue Y92 towards Her2 of the four variants were estimated using solvent accessible surface area (SASA) by NACCESS. The discrepancy of SASA Y92 between the mutants and WT were confirmed by a two tailed T-test: p-values < 0.0005 with 95% confidence, indicating that the interacting residue Y92 (on Vκ-CDR3) is noticeably hidden towards the Vκ-FWR3 in the delTE mutant when compared to the others, thereby diminishing its binding to Her2.
